# Resource Recovery from High-Performance Textile Waste: Carbon Footprint Assessment, Graded Recycling, and Product Development Pathway for Used Firefighting Protective Clothing

**DOI:** 10.3390/ma19061188

**Published:** 2026-03-18

**Authors:** Xing Zhang, Zhenhao Sun, Xiaoxian Wang, Jingru Lu, Hu Gu, Hongjing Zhong, Xiaoyun Long, Qilong Sun, Wei Ye

**Affiliations:** 1 National & Local Joint Engineering Research Center of Technical Fiber Composites for Safety and Protection, Nantong University, Nantong 226019, China; zx0211@ntu.edu.cn (X.Z.); lxy1988@ntu.edu.cn (X.L.); 2 College of Textiles and Clothing, Nantong University, Nantong 226019, China; 2415110053@stmail.ntu.edu.cn (Z.S.); 2415110331@stmail.ntu.edu.cn (X.W.); 2415110328@stmail.ntu.edu.cn (J.L.); 2315110049@stmail.ntu.edu.cn (H.Z.); 3 Hangzhou Institute of Quality & Metrology, Hangzhou 310069, China; guh@hzzjy.net

**Keywords:** protective clothing, aramid fiber, circular utilization, carbon footprint, regenerative design, gradient utilization

## Abstract

**Highlights:**

- Filling the research gap in recycling high-performance aramid fibers from specialized waste streams.
- First quantifies the full lifecycle carbon footprint of firefighting clothing (89.03–102.29 kg CO_2_-eq/suit).
- A novel three-tier recycling model achieves near 100% material recovery with low processing emissions (4.6 kg CO_2_-eq/suit).
- Integrating cultural heritage and digital passports enhances the market value and acceptance of recycled products.
- Presents a scalable integrated system for the assessment, design, and traceability of high-performance textile recycling.

**Abstract:**

The global textile industry, challenged by resource depletion and environmental pollution, urgently requires a shift toward a circular economy. However, recycling efforts remain limited, focusing mainly on conventional fibers and neglecting high-performance materials like aramid. This study addresses the recycling of used firefighting protective clothing-an aramid-rich, high-turnover waste stream typically landfilled or incinerated. Life cycle assessment reveals the significant carbon footprint of its production and disposal, underscoring the need for circular strategies. A systematic recycling framework is established, integrating collection logistics and redesign principles. A graded “three-tier” approach is proposed, enabling direct reuse, yarn regeneration, and non-woven production based on material conditions. High-value products were developed by incorporating firefighting heritage and intangible cultural crafts into the design, supported by digital product passports for traceability. These strategies enhanced market acceptance and emotional value. The work provides a scalable circular solution for high-performance textiles, aiming to extend material life, reduce carbon emissions, and advance sustainable textile management through a novel combination of technical recycling and cultural value addition.

## 1. Introduction

This study addresses the recycling of used firefighting protective clothing—an aramid-rich, high-turnover waste stream typically landfilled or incinerated. Life cycle assessment reveals the significant carbon footprint of its production and disposal, underscoring the need for circular strategies. We therefore establish a systematic recycling framework integrating collection logistics and redesign principles, proposing a graded “three-tier” approach that enables direct reuse, yarn regeneration, and non-woven production based on material conditions. To enhance market acceptance and emotional value, we incorporate firefighting heritage and intangible cultural crafts into product design, supported by Digital Product Passports for traceability. This work aims to provide a scalable circular solution for high-performance textiles, extending material life and reducing emissions through a novel combination of technical recycling and cultural value addition.

The urgency of this research is underscored by the global textile industry’s sustainability challenges. The prevailing linear “take-make-dispose” model not only shortens textile lifespans but also imposes severe environmental costs [1,2,3]. While “fiber-to-fiber” closed-loop recycling is recognized as a key pathway toward circular economy [4,5], current progress remains limited: the global “textile-to-textile” recycling rate is only approximately 1%, with most recycled fibers originating from plastic bottles rather than post-consumer textiles. As a regional reference, China’s policy aims to achieve an annual recycled fiber output of 2 million tons from waste textiles by 2025, though this is a target, not actual global production data [6,7], highlighting significant gaps in the current textile circularity system [1,8]. Existing recycling technologies primarily focus on conventional fibers like cotton and polyester, while high-performance fibers such as aramid remain largely underexplored [9,10,11]. Given that high-performance fibers typically cost 10–20 times more than conventional counterparts, their recovery presents strong economic incentives alongside environmental benefits [2]. Recent policy developments, including the EU’s Digital Product Passport initiative and China’s waste uniform recycling directives, create a favorable regulatory environment for addressing this gap [7,12].

Research indicates that the global market for firefighting apparel is approximately USD 1.9 billion, with structural firefighting protective clothing (SFPC) as the primary category, accounting for over 50% of the value and showing sustained growth. Concurrently, SFPC has a high turnover rate; for instance, in China, the average replacement cycle is about three years, meaning hundreds of millions of dollars worth of SFPC are retired annually [13]. As inherently flame-retardant garments, particularly those made from aramid fibers, they take over 100 years to degrade in landfills. They are unsuitable for waste-to-energy incineration, as the process may release significant quantities of toxic and hazardous gases, causing severe environmental pollution [14]. Despite gradual improvements in the policy landscape, practical recycling of waste SFPC faces multiple challenges. For example, in carbon footprint research for textiles, while studies have assessed the production stages of common fibers like silk and cotton [15], a unified carbon accounting methodology for complex blends and functional garments is still lacking.

At the technological level, current research on aramid fiber recycling primarily follows two paths: physical and chemical recycling. Physical recycling involves sorting, cleaning, and mechanical processing to transform waste SFPC into cultural products or short-fiber reinforcements [16,17]. Chemical recycling employs novel solvents or catalytic depolymerization to break down fibers into monomers for high-value regeneration [18,19]. However, both face industrialization hurdles: the physical recycling value chain is underdeveloped, lacking systematic research on waste garment utilization rates, fiber property retention, product design, and market outreach; chemical methods are constrained by complex processes, high solvent costs, and significant energy consumption [5,20,21]. Regarding collection systems, although several regional textile recycling clusters have formed in China, the overall focus remains on low-value-added refurbishment, lacking specialized networks for high-performance textiles. This results in a significant portion of waste SFPC failing to enter effective resource recovery channels [22,23]. In the market, awareness and acceptance of recycled safety textiles need improvement. Currently, only a few international companies have engaged in related practices, such as reprocessing aramid waste into new yarns for cut-resistant gloves and flame-retardant fabrics [24], or transforming firefighting suit materials into new textiles [25]. Systematic product development has yet to emerge. Future efforts need to integrate design and cultural elements to further enhance product added value and market acceptance.

## 2. Materials and Methods

This study systematically investigates the circular utilization of waste firefighting protective clothing. First, the entire lifecycle-encompassing production, use, and disposal-is systematically traced through lifecycle analysis. Subsequently, based on existing carbon footprint assessment methods for textiles, carbon emissions from the production and disposal stages are quantified. Building on this, a recycling framework for waste firefighting protective clothing is established, and the carbon footprints of different circular pathways are calculated. Following this, circular recycling technology routes for waste garments are systematically summarized, along with practical development of recycled products. Finally, integrating market feedback and challenges encountered in circular practice, a recycling and regeneration design strategy based on the “three-tier gradient” principle is proposed, providing a theoretical foundation for the resource utilization of functional textiles.

### 2.1. Lifecycle of Firefighting Protective Clothing

**Figure 1:**
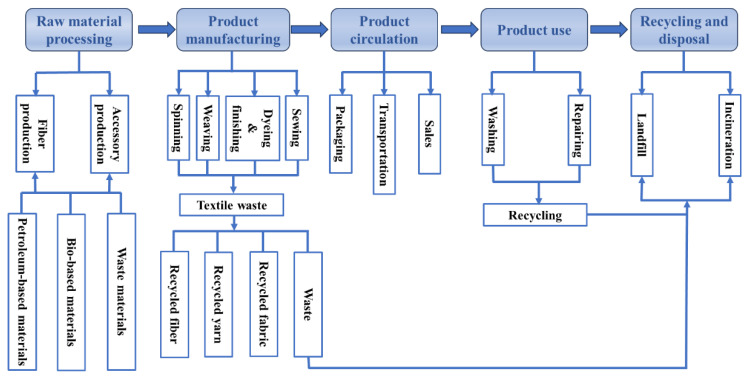
Lifecycle diagram of firefighting protective clothing.

Similarly to general textiles, the lifecycle of firefighting protective clothing encompasses raw material production, product manufacturing, packaging and transportation, sales, use, and final disposal. In response to the current lack of a recycling system for such garments, this study systematically analyzes their lifecycle pathways through questionnaires and field research, and develops a lifecycle diagram of firefighting protective clothing under existing market conditions (Figure 1).

The lifecycle of firefighting protective clothing can be divided into five main stages:

The first stage is raw material production, involving the production of various fibers and garment accessories.

The second stage is product manufacturing, including processes such as spinning, weaving, dyeing and finishing, and garment assembly.

The third stage is product distribution, covering packaging, transportation, and sales.

The fourth stage is product use, primarily involving maintenance activities such as washing and repair.

The fifth stage is waste treatment, with research indicating that most used firefighting protective clothing is currently landfilled or incinerated after collection [26].

Given that the primary material of firefighting protective clothing is high-performance fibers such as aramid, which is difficult to degrade naturally and unsuitable for energy recovery [27], their treatment processes tend to generate significant pollutants [28,29]. As a result, solid waste represents the most notable environmental impact factor in their lifecycle. Promoting the recycling of used firefighting protective clothing is an effective approach to mitigating its solid waste issue. Furthermore, studies on the carbon footprint of textiles indicate that carbon emissions during the clothing lifecycle are concentrated in the raw material production, product manufacturing, and consumer use stages [30], while waste recycling can significantly reduce resource consumption, highlighting the importance of recycling used garments.

### 2.2. Material Analysis

Firefighting protective clothing is widely deployed by fire and rescue departments, regional micro fire stations, and civilian rescue organizations. It typically consists of a jacket and pants, structured with an outer shell, a middle layer (comprising a thermal barrier and a waterproof, moisture-permeable layer), and an inner comfort layer, along with various accessory components. Manufactured by Taizhou Sanjiang Fire Fighting Equipment Co., Ltd., Taizhou, Jiangsu, China. The specific material composition is as follows:

- Outer Shell Fabric: 98% aramid fiber, 2% conductive yarn.
- Middle Layer: The thermal barrier is 100% aramid fiber, and the waterproof, moisture-permeable layer is a polytetrafluoroethylene (PTFE) membrane.
- Comfort Layer: 50% aramid fiber, 50% flame-retardant viscose.
- Accessories: Zippers and hook-and-loop fasteners are made of flame-retardant nylon; the waist belt is 100% aramid fiber; metal buckles are 100% steel; and the sewing thread is also 100% aramid fiber.

**Figure 2:**
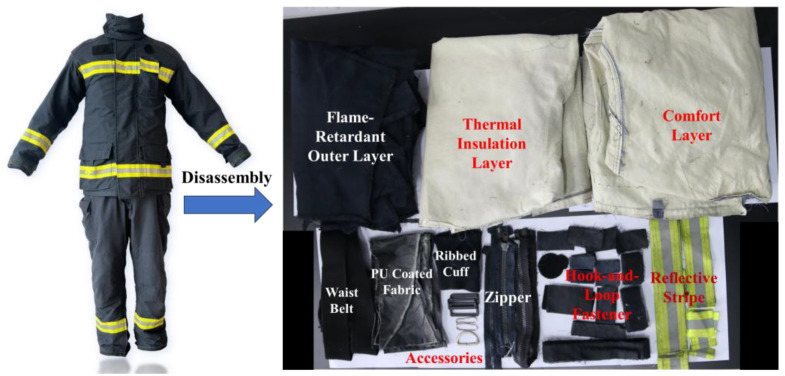
Overview and component breakdown of the garment.

An overview of the complete garment and a schematic breakdown of its components are shown in Figure 2.

### 2.3. Carbon Footprint Assessment

According to research data, China had approximately 500,000 full-time and part-time fire and rescue personnel and over 500,000 micro fire stations in 2024. On average, each firefighter is equipped with more than two sets of firefighting protective clothing, and each micro fire station is equipped with more than three sets. This results in a total national inventory of roughly 2.5 million sets. With an estimated annual retirement rate of 20%, about 500,000 sets of discarded firefighting protective clothing require disposal each year. Given a procurement cost exceeding CNY 2000 per set, the annual value of discarded clothing awaiting treatment surpasses one billion yuan. Currently, these garments are primarily disposed of via landfill or incineration, leading to significant resource waste and environmental pollution.

To address this, this study aims to achieve the circular reuse of used firefighting protective clothing by establishing a systematic recycling system and conducts a carbon footprint accounting for different recycling pathways. Carbon footprint refers to the total greenhouse gas (primarily carbon dioxide) emissions generated throughout a product’s entire lifecycle, expressed in carbon dioxide equivalent (CO_2_-eq) [31]. It serves as a key indicator for assessing sustainable development performance. Through carbon footprint assessment, emission hotspots across various lifecycle stages can be systematically identified, providing a basis for formulating targeted emission reduction strategies.

When conducting carbon footprint assessment and analysis, it is first necessary to clarify the distribution of carbon emissions throughout the entire lifecycle of the target product. This study divides the lifecycle of firefighting protective clothing into three stages: raw material production, finished product processing, and post-use treatment (as shown in Table 1). This framework encompasses the entire process from fiber preparation to final disposal, enabling a comprehensive assessment of its carbon footprint characteristics.

The analysis reveals significant differences in carbon emissions across the various lifecycle stages of firefighting protective clothing. It should be noted that a degree of uncertainty exists in the carbon footprint accounting process, and different calculation methods may lead to data discrepancies [32]. The specific carbon emission reference data for each stage are as follows (emissions per kg unless otherwise specified):

- Raw Material Production Stage: Aramid fiber: 17.9 kg CO_2_-eq [33]; Polytetrafluoroethylene (PTFE): 5.9 kg CO_2_-eq [34]; Flame-retardant viscose fiber: 3.8 kg CO_2_-eq [35]; Nylon fiber: 6.5 kg CO_2_-eq [35]; Nylon resin: 3.43 kg CO_2_-eq [36]; Steel: 1.8 kg CO_2_-eq [37]. Polyurethane foam: 2.95–7.67 kg CO_2_-eq [38]. The low-carbon coating process currently used generates emissions of 0.6 kg CO_2_-eq per square meter.
- Product Manufacturing Stage: Spinning: 2.8–4.56 kg CO_2_-eq [39]; Weaving: 1.43–3.29 kg CO_2_-eq [40]; Fabric dyeing: 8.8 kg CO_2_-eq [39]; Garment making (including cutting, sewing, printing, finishing, ironing, packaging, and auxiliary processes such as lighting, air conditioning, and ventilation): 2.07–6.93 kg CO_2_-eq [40,41].
- Waste Treatment Stage: As the waste is unsuitable for energy recovery, only standard incineration is considered, with emissions ranging from 2.03 to 5.89 kg CO_2_-eq [42]. If directly landfilled, emissions are 0.42 kg CO_2_-eq [43].

Several clarifications are necessary for this assessment. The Waist Belt woven fabric used in the garment does not involve a separate spinning process, and related accessories require no further machining. Furthermore, the thermal barrier layer retains its original color and undergoes no dyeing treatment. Carbon emission data for the forming processes of the PTFE membrane and certain accessories are not explicitly available in the existing literature; given their minimal contribution to the overall mass, these are neglected in this study’s calculations. Considering the greater processing difficulty of high-performance fibers like aramid, the relatively heavy and complex structure of the garment, and its inherent flame-retardant properties, the upper bounds of the emission ranges were uniformly adopted for this analysis. Additionally, due to the flame-retardant nature of the materials, incineration emissions are set to the maximum value within the range. Metallic accessories are assumed not to undergo incineration or landfilling.

### 2.4. Construction of the Circular Utilization System

The circular utilization system for products primarily consists of two core components: collection and regeneration. In this study, these are defined, respectively, as the “Collection Model for Used Firefighting Protective Clothing” and the “Regenerative Design of Firefighting Protective Clothing,” which together form a closed-loop circular pathway.

#### 2.4.1. Establishment of the Collection Model

**Figure 3:**
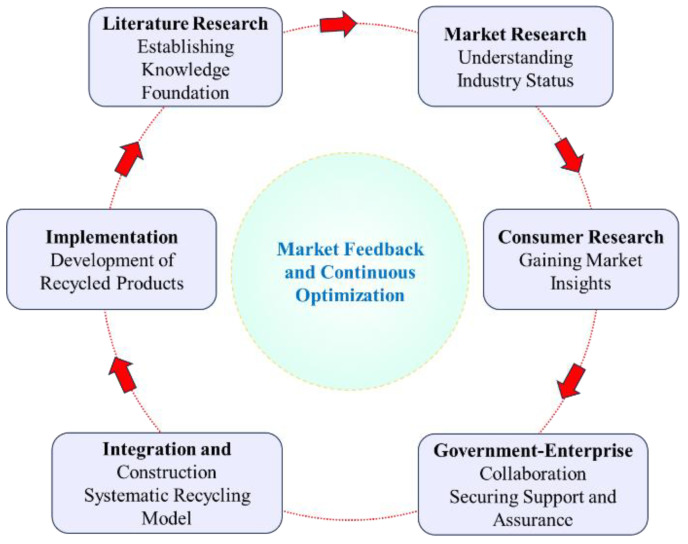
Flowchart of the collection model construction and implementation process.

Given that firefighting protective clothing constitutes specialized professional attire, its collection process possesses unique characteristics. Therefore, prior to constructing the collection system, it is necessary to conduct systematic preliminary research to comprehensively assess the current market status of collection, reuse potential, and technical-economic feasibility. The specific implementation process is illustrated in Figure 3: First, a literature review is conducted to outline the domestic and international status of treatment methods and technological pathways for used firefighting protective clothing. Second, market research is carried out by visiting production units and user departments to clarify material composition, retirement mechanisms, and market inventory. Furthermore, consumer research is performed to gather public opinion and acceptance regarding clothing recycling and recycled product development. Subsequently, engagement with industry associations and government agencies is pursued to secure policy and resource support. Finally, based on the integration of findings from these multifaceted research efforts, a systematic collection model for firefighting protective clothing is constructed, and the development and implementation of related recycled products are promoted.

#### 2.4.2. Development of Recycled Products

Textile waste can be managed through various approaches, which, according to existing research, are categorized into four types [44]:

(i) Primary recycling, which involves the direct or minimally processed reuse of waste, often applicable to pre-consumer waste;

(ii) Secondary recycling, where fibers are recovered through physical methods and reprocessed into recycled products with properties close to those of the original material;

(iii) Tertiary recycling, which employs pyrolysis or chemical depolymerization to degrade fibers into monomers for regeneration;

And (iv) Quaternary recycling, where energy is recovered through incineration or landfill gas capture.

**Figure 4:**
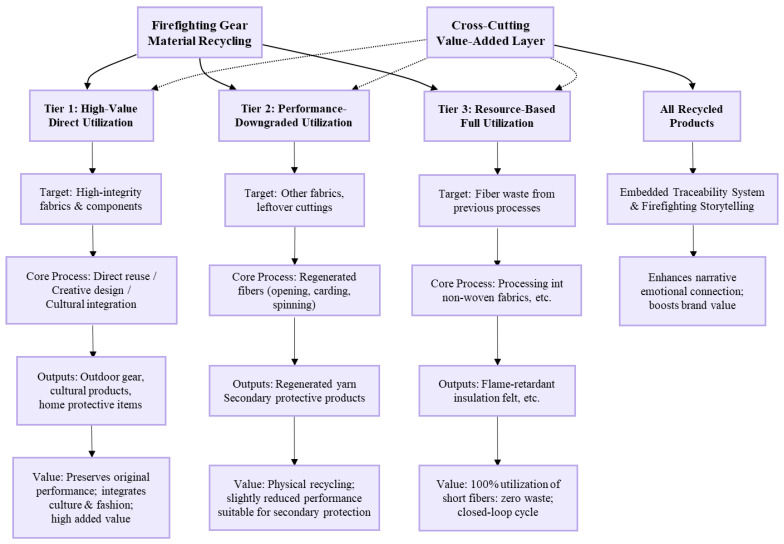
Sorted and graded reuse model.

Currently, tertiary recycling technologies remain underdeveloped. Therefore, this study primarily focuses on primary and secondary recycling pathways. Based on the material condition and integrity of the garments, a graded reuse model has been established, incorporating both sorting and tiered processing (as shown in Figure 4).

The model consists of three gradient directions:

First, fabrics and accessories in good condition are directly reused. By integrating fashion and cultural elements, new, high-value-added products are developed.

Second, other fabrics and leftover cuttings from trimming undergo regenerated spinning to produce yarns for secondary protective products.

Finally, fiber waste generated in preceding processes is processed into non-woven fabrics, which are further developed into thermal insulation products.

Throughout the recycled product development process, a traceability information system will be implemented to convey the spirit of firefighting culture, thereby enhancing product narrative and public recognition.

## 3. Results

### 3.1. Carbon Footprint Calculation for the Circular Utilization

**Figure 5:**
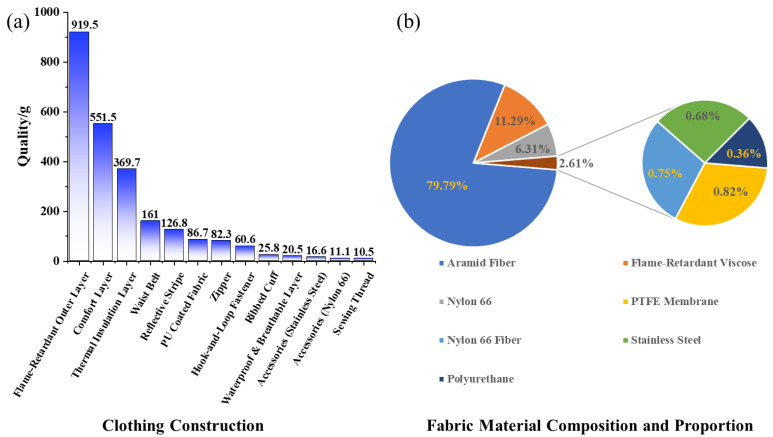
Structural and material analysis of firefighting protective clothing: (**a**) weight distribution of garment components; (**b**) material composition and proportions.

Based on Table 1, the firefighting protective clothing was disassembled into 13 components, and the weight of each was summarized (Figure 5a). Concurrently, an analysis of the garment’s constituent materials was conducted (Table 1), with the specific material composition percentages shown in Figure 5b. Using the aforementioned parameters, and applying Formulas (1) and (2), the total carbon emissions from the production and end-of-life treatment stages for a single firefighting protective clothing weighing 2.44 kg were calculated (A complete set of used firefighting protective clothing, including the jacket and trousers). The total emissions amount to 102.29 kg CO_2_-eq if incinerated and 89.03 kg CO_2_-eq if landfilled.

(1) $$E_{total}^{incin}=\sum_{j=1}^{m}M_{j}\cdot \left(EP_{j}+EI_{j}\right)+W\cdot EC$$

(2) $$E_{total}^{landfill}=\sum_{j=1}^{m}M_{j}\cdot \left(EP_{j}+EL_{j}\right)+W\cdot EC$$

where *m* is the number of material types; *j* is the material index (*j* = 1, 2, …, *m*); *M_j_* is the total mass of material *j* in the garment (kg); *EP_j_* is the production emission factor for material *j* (kg CO_2_-eq/kg, covering the process from raw material to fiber); *EI_j_* is the incineration emission factor for material *j* (kg CO_2_-eq/kg); *EL_j_* is the landfill emission factor for material *j* (kg CO_2_-eq/kg); *W* is the total weight of the garment (kg); *EC* is the garment manufacturing emission factor (kg CO_2_-eq/kg, representing the processing from materials to finished garment, assumed proportional to total weight).

Through the disassembly analysis of used suits and guided by the three-tier gradient reuse principle, the garments were categorized into four groups: outer shell fabric, thermal barrier layer, comfort layer, and other accessories. After processing, four types of recycled products can be obtained: fabric, yarn, non-woven fabric, and accessories. To quantify the output per used suit, the following utilization rates were established:

Tier 1 (Recycled Fabric):

~70% of the outer shell fabric is directly usable as recycled fabric; the remaining 30% enters Tier 2.

~95% of the thermal barrier layer is directly usable as recycled fabric; the remaining 5% enters Tier 2.

~85% of the comfort layer is directly usable as recycled fabric; the remaining 15% enters Tier 2.

Tier 2 (Feedstock for Recycled Yarn):

30% from the outer shell.

5% from the thermal barrier.

15% from the comfort layer.

100% of the sewing thread.

100% of the ribbed cuffs.

Tier 3 (Feedstock for Recycled Non-woven Fabric):

~30% waste generated collectively during the opening, carding, and spinning processes for yarn production.

100% of the PU-coated fabric.

10% of the reflective tape.

Recycled Accessories:

Accessories: 100% intact.

Waist belt: 100% intact.

Zippers: ~100% intact.

Reflective tape: ~90% intact.

Hook-and-loop fasteners: ~80% intact.

Based on this graded utilization pathway, the following formulas were established to calculate the output of recycled products:

Tier 1: Output of Recycled Fabric

(3) $$M_{fabric}=w_{o}\cdot \alpha_{o}+w_{i}\cdot \alpha_{i}+w_{c}\alpha_{c}$$

Tier 2: Input for Recycled Yarn Production

(4) $$M_{yarn}=w_{o}\left(1-\alpha_{o}\right)+w_{i}\left(1-\alpha_{i}\right)+w_{c}\left(1-\alpha_{c}\right)+w_{s}+w_{t}$$

Tier 3: Input for Recycled Non-woven Fabric Production

(5) $$M_{nonwoven}=M_{yarn}\cdot \eta +w_{p}+w_{r}\cdot 10\%$$

where *w_o_*, *w_i_*, *w_c_* are the weights (kg) of the outer shell, thermal barrier, and comfort layer, respectively; *w_s_*, *w_t_* are the weights (kg) of the sewing thread and ribbed cuffs, respectively; *w_p_*, *w_r_* are the weights (kg) of the PU-coated fabric and reflective tape, respectively; *α_o_* = 70%, *α_i_* = 95%, *α_c_* = 85% represent the proportion of each layer directly used as recycled fabric; *η* = 30% is the waste rate during the recycled yarn preparation process.

**Figure 6:**
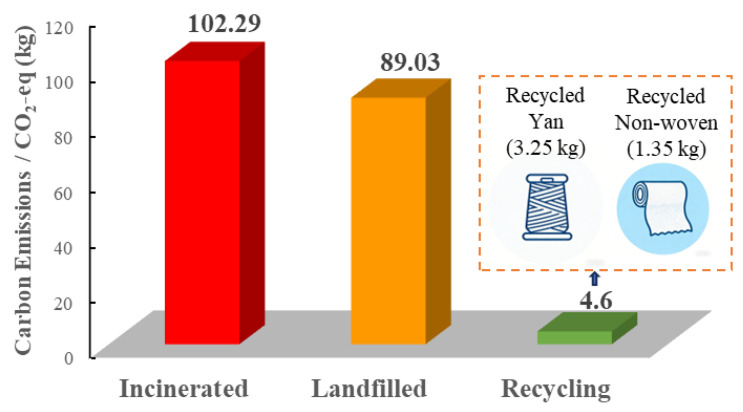
Carbon Emission Situation of Recycling and Reutilization of Used Firefighting Protective Clothing.

Applying Formulas (3)–(5) and the component weights from Figure 5a, the calculation shows that one used firefighting protective clothing weighing 2.44 kg can yield 1.46 kg of recycled fabric, 0.29 kg of feedstock for recycled yarn, and 0.22 kg of feedstock for recycled non-woven fabric. The spinning and non-woven manufacturing processes incur new carbon emissions, as detailed in Table 2. The calculations indicate emissions of 3.25 kg CO_2_-eq for the recycled yarn preparation and 1.35 kg CO_2_-eq for the recycled non-woven fabric preparation per suit (Figure 6). The recycled fabric and accessories involve direct reuse and thus no processing emissions. The end-of-life emissions for partially damaged hook-and-loop fasteners are not included due to their negligible mass.

Therefore, while the total carbon emissions for a single used firefighting protective clothing (2.44 kg) across production and disposal amount to at least 89.03 kg CO_2_-eq (landfill scenario), the proposed graded three-tier circular model generates only 4.6 kg CO_2_-eq in processing emissions, thereby significantly extending its lifecycle. This finding not only validates the technical feasibility of this circular model but also holds profound practical significance. If this model were scaled up to address the millions of firefighting suits discarded annually nationwide or even globally, it could potentially reduce carbon emissions by tens of thousands of tons of CO_2_ equivalent—an environmental benefit comparable to the annual carbon sequestration of hundreds of thousands of trees. Compared with current mainstream physical recycling (which typically downgrades fibers to low-value fillers) and chemical recycling (which often faces industrialization bottlenecks due to high energy consumption and costs), the graded utilization model proposed in this study maximizes the preservation of original fiber value while achieving carbon reduction. This provides a valuable reference for the circular utilization of safety and protective textiles, offering both environmental and economic benefits.

The circular utilization of retired firefighting protective clothing has achieved broad societal consensus, supported by a solid technical foundation and promising market prospects for resource recovery. Research confirms that aramid, the core material, retains excellent mechanical properties and flame retardancy after decommissioning, significantly outperforming conventional textile materials and demonstrating considerable potential for high-value applications [26]. Other components, such as flame-retardant viscose fibers and accessories, also preserve their inherent protective properties, maintaining application value in safety-related fields. Based on the above analysis and the sorted graded reuse model illustrated in Figure 4, this study proposes a graded “three-tier” utilization framework based on the principles of fabric, yarn, and non-woven recycling, this model employs fabric re-engineering and fiber regeneration as core technologies to establish a zero-waste circular utilization system, achieving near-complete recovery of primary functional materials, with ancillary materials and processing losses also being valorized through pathways such as non-woven production, resulting in an overall material recovery rate of nearly 100% by mass.

Compared with current mainstream physical and chemical recycling methods, the proposed “three-tier” model demonstrates clear advantages in fiber value retention and carbon emission control. Physical recycling, while simple and low-cost, typically downgrades high-performance fibers into low-value fillers or chopped fibers, making closed-loop recycling difficult to achieve. Chemical recycling can regenerate monomers through depolymerization, theoretically restoring original fiber properties; however, its industrialization remains constrained by complex processes, high energy consumption, and expensive solvents, limiting large-scale application. In contrast, the graded utilization model proposed in this study adapts to material conditions, maximizing the preservation of original fiber value while achieving carbon reduction. This provides a feasible pathway for the circular utilization of high-performance textiles that balances both environmental and economic benefits.

Garments collected for recycling require systematic evaluation across three dimensions: hygiene and safety, appearance integrity, and material performance. Evaluation prioritizes hygiene and safety, strictly excluding garments exposed to toxic or hazardous substances. On this basis, the assessment further examines appearance characteristics such as wear level and color retention, and includes experimental testing for mechanical strength and flame-retardant performance. This process establishes a scientific material grading standard. The evaluation mechanism ensures the quality of recycled products in terms of safety, performance, and esthetics. It provides a precise raw material classification basis for subsequent product development, facilitating the standardized transformation from used equipment to regulated recycled feedstock.

### 3.2. Recycling Product Design and High-Value Development

**Figure 7:**
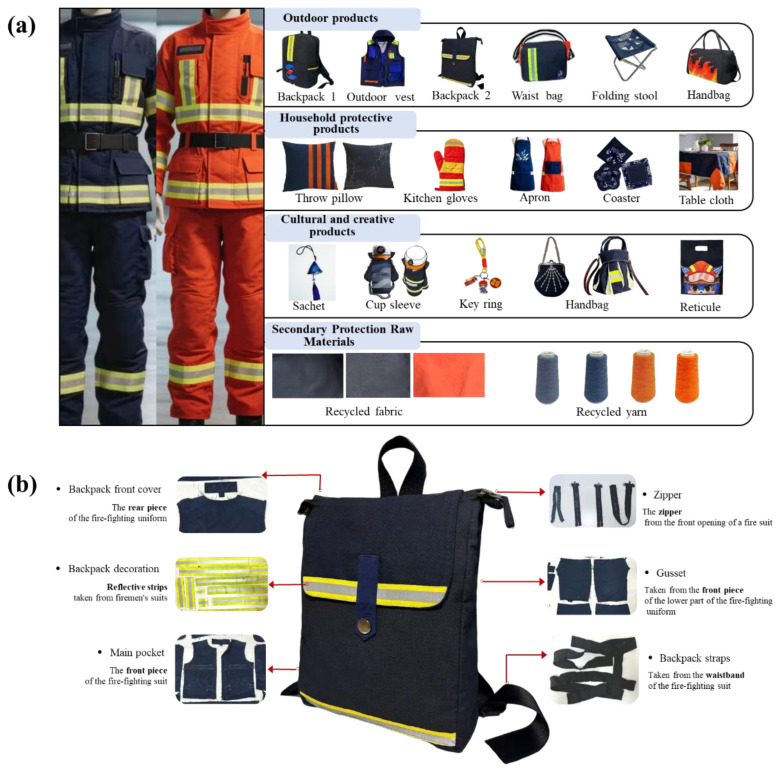
Recycled Design from Used Firefighting Equipment: (**a**) Design of the circular product series; (**b**) Composition diagram of the recycled components for Backpack 2.

In the recycling of used firefighting protective clothing, we have established an integrated product development system encompassing assessment, design, and digitalization. A series of products developed utilizing retired firefighting equipment (including structural firefighting gear and wildland firefighting clothing) are shown in Figure 7.

Following collection and systematic assessment [26], the used clothing is categorized into three main types of recycled materials: recycled fabrics, recycled yarns, and recycled non-woven materials. Original garment accessories are also directly reused in the designs.

For fabrics in good condition, their inherent performance advantages-such as flame retardancy, abrasion resistance, and tear resistance-are fully leveraged to develop products like outdoor gear, household protective items, secondary protective products, and cultural merchandise (Figure 7a). These designs actively incorporate original accessories (Figure 7b).

For recycled yarns, the blend ratio of recycled fibers can be adjusted, or they can be blended with other colored flame-retardant fibers to meet diverse customer needs. This type of yarn can also be used to produce higher-grade recycled fabrics.

For non-woven materials, various products like flame-retardant insulating felts can be developed, which can subsequently be integrated into finished products made from the first-tier recycled fabrics.

To enhance product value, this study integrates firefighting elements with traditional Chinese cultural symbols and intangible cultural heritage (ICH) crafts, boosting market recognition and acceptance of the recycled products. For instance, the design team created a series of textile products inspired by traditional mythical beasts, infusing cultural significance while preserving material functionality. Additionally, explorations into combining ICH techniques like tie-dyeing and embroidery with recycled fabrics have yielded protective yet artistic handicrafts, expanding their market potential into areas like gifts and promotional items.

Within the EU’s sustainable textiles policy framework, the DPP is identified as a key tool for achieving circularity goals [11]. This system uses electronic identifiers to record and provide core information such as product composition, care/repair guidelines, and recycling instructions. It aims to build consumer trust through transparency and offers data support for professional sorting and high-value recycling of end-of-life products, thereby promoting standardized operations across the recycling industry chain. This study has also designed a traceability management system, equipping each recycled product with a digital passport that records its composition and origin. This enhances consumer trust and fosters an emotional connection. Table 3 shows the traceability information for Backpack 2. This digital system can enables full-process traceability from the “frontline of rescue” to the “consumer market,” ensuring product quality control while also strengthening the brand’s emotional value and cultural narrative by sharing the stories behind the firefighting gear.

The circular recycling of used firefighting protective clothing represents not merely a physical transformation of materials but a multi-dimensional reshaping of function, culture, and emotion. Through systematic assessment, innovative design, cultural integration, and digital empowerment, used equipment is transformed into high-value daily protective products and cultural carriers. This achieves a synergy between resource circulation and value enhancement, providing a practical pathway for promoting green consumption and fostering a culture of safety.

### 3.3. Potential Limitations

The proposed three-tier model has certain limitations. First, its effectiveness heavily depends on accurate sorting and material assessment of retired firefighting suits. In practice, varying degrees of contamination, thermal aging, or mechanical damage during use can lead to inconsistent fiber degradation, potentially affecting the “direct reuse” and “yarn regeneration” tiers. Additionally, the model was developed specifically for firefighting protective clothing; its applicability to other high-performance textiles (e.g., industrial protective wear, aerospace composites) requires further validation.

Second, from an LCA methodology perspective, several stages were excluded from the system boundary due to data constraints: transportation emissions (excluded due to high uncertainty in collection pathways and distances); use phase emissions (excluded due to lack of unified washing and maintenance benchmarks); infrastructure impacts (excluded following ISO 14044 standards [48] and mainstream LCA practices); and collection logistics (excluded due to decentralized collection networks, warranting future regional studies).

Consequently, the reported 4.6 kg CO_2_-eq per suit represents only direct processing emissions from the recycling stage, excluding broader system impacts. Future research should incorporate these omitted stages as more standardized data become available.

### 3.4. Economic, Logistical, and Regulatory Challenges for Practical Implementation

From an economic perspective, although the three-tier model offers advantages in carbon emissions, its large-scale promotion still faces challenges such as high initial investment and difficulties in controlling recycling logistics costs. In particular, the “yarn regeneration” tier involves multiple steps including disassembly, cleaning, sorting, and spinning. The current lack of mature automated disassembly equipment results in relatively high labor costs.

From a logistical perspective, a systematic collection network for used firefighting protective clothing has yet to be established. Currently, firefighting suits are primarily managed by individual fire brigades in a decentralized manner, lacking unified recycling channels and reverse logistics systems, leading to low collection efficiency and high transportation costs.

From a regulatory perspective, although the EU’s EPR scheme and relevant Chinese policies provide policy support for high-performance textile recycling, specialized recycling standards for used firefighting protective clothing, certification systems for recycled materials, and the practical implementation of Digital Product Passports remain in early stages, lacking mandatory recycling targets and market access mechanisms.

## 4. Conclusions

This study addresses a critical research gap by establishing a circular utilization framework for retired firefighting protective clothing, one of the few specialized waste streams rich in high-performance aramid fibers. Through lifecycle assessment, we provide the first empirical quantification of the carbon footprint of firefighting suits, revealing that traditional disposal methods (landfill or incineration) generate 89.03–102.29 kg CO_2_-eq per suit. In response, we propose a novel three-tier gradient recycling model—encompassing direct reuse, yarn regeneration, and non-woven production—which achieves near 100% material recovery while adding only 4.6 kg CO_2_-eq in processing emissions per suit. By integrating cultural heritage narratives and intangible crafts into product redesign, and leveraging Digital Product Passports (DPP) for traceability, the model significantly enhances the market acceptance and emotional value of recycled products. This work presents a scalable, integrated system for the assessment, design, and traceability of high-performance textile recycling, offering a transformative pathway toward sustainable waste management in the protective clothing sector.

## Figures and Tables

**Table 1:** Carbon emissions across the full lifecycle of firefighting protective clothing.

Clothing Construction	Material	Blend Ratio	CO_2_-eq (kg)/per Kilogram (by Production Stage)		
			Raw Material Production	Spinning	Weaving
Flame-Retardant Outer Layer	Aramid Fiber	98%	17.9	2.8–4.56	1.43–3.29
	Nylon 66 Conductive Yarn	2%	6.5	2.8–4.56	1.43–3.29
Thermal Insulation Layer	Aramid Fiber	100%	17.9	2.8–4.56	1.43–3.29
Comfort Layer	Flame-Retardant Viscose	50%	3.8	2.8–4.56	1.43–3.29
	Aramid Fiber	50%	17.9	2.8–4.56	1.43–3.29
Waist Belt	Aramid Fiber	100%	17.9	2.8–4.56	1.43–3.29
Sewing Thread	Aramid Fiber	100%	17.9	2.8–4.56	1.43–3.29
Reflective Stripe	Aramid Fiber	100%	17.9	2.8–4.56	1.43–3.29
PU Coated Fabric	Aramid Fiber	90%	17.9	2.8–4.56	1.43–3.29
	Polyurethane	10%	2.95–7.67	/	/
Ribbed Cuff	Aramid Fiber	100%	17.9	2.8–4.56	1.43–3.29
Waterproof and Breathable Layer	PTFE Membrane	100%	5.9	/	/
Zipper	Flame-Retardant Nylon 66	100%	3.43	/	/
Hook-and-Loop Fastener	Flame-Retardant Nylon 66	100%	3.43	/	/
Accessories (Resin)	Flame-Retardant Nylon 66	100%	3.43	/	/
Accessories (Metal)	Stainless Steel	100%	1.8	/	/

Note: The “/” in the table indicates that this processing step is not involved.

**Table 2:** Carbon footprint in the processing of recycled products from used firefighting protective clothing.

Category	Recycled Yarn	Recycled Non-Woven Fabric
Preparation Process	1. Sterilization, Cleaning, and Drying. 2. Opening and Loosening 3. Spinning 4. Weaving (additional step)	1. Sterilization, Cleaning, and Drying. 2. Opening, Blending and Carding 3. Web Forming 4. Needle Punching
Carbon Emissions (CO_2_-eq (kg)/per kilogram)	1. Cleaning Treatment: 1.90 [45] 2. Opening and Loosening: 1.44 [46] 3. Spinning: 4.56 [39] 4. Weaving: 3.29 [40]	Total: 6.14 [40,45]

**Table 3:** Traceability Information for Backpack 2.

Category	Specific Information
Product Identity Identification	ID: 20251225BB001.
	Date: 25 December 2025.
Material Composition and Source Traceability	100% recycled material from ued firefighting protective clothing.
	Outer: 98% Aramid and 2% Conductive Nylon.
	Inner: 50% Aramid and 50% FR Viscose.
	Shoulder Straps: 100% Aramid.
	Reflective Stripe: 100% Aramid
	Zippers and Hooks: 100% Flame Retardant Nylon.
Product Performance and Safety Information	Possesses flame retardant, wear-resistant, and cut-resistant properties.
	Complies with the European standard EN 12927 [47].
Recycling and Circularity Guidance	Please contact us for professional recycling upon product end-of-life.
	Potential Next Life Cycle: Recycled yarn and non-woven fabrics.
Story and Emotional Connection	Raw materials sourced from used protective clothing of the Nantong Fire Brigade, which participated in 3 fire rescue operations.
	Incorporates intangible cultural heritage techniques such as Nantong Blue Calico.
Manufacturer	Nantong University.

## Data Availability

The original contributions presented in this study are included in the article. Further inquiries can be directed to the corresponding authors.
